# Microenvironmental Regulation by Fibrillin-1

**DOI:** 10.1371/journal.pgen.1002425

**Published:** 2012-01-05

**Authors:** Gerhard Sengle, Ko Tsutsui, Douglas R. Keene, Sara F. Tufa, Eric J. Carlson, Noe L. Charbonneau, Robert N. Ono, Takako Sasaki, Mary K. Wirtz, John R. Samples, Liselotte I. Fessler, John H. Fessler, Kiyotoshi Sekiguchi, Susan J. Hayflick, Lynn Y. Sakai

**Affiliations:** 1 Department of Biochemistry and Molecular Biology, Oregon Health and Science University, Portland, Oregon, United States of America; 2 Shriners Hospital for Children, Portland, Oregon, United States of America; 3 Laboratory of Extracellular Matrix Biochemistry, Institute for Protein Research, Osaka University, Osaka, Japan; 4 Casey Eye Institute, Department of Ophthalmology, Oregon Health and Science University, Portland, Oregon, United States of America; 5 Department of Molecular, Cell, and Developmental Biology and Molecular Biology Institute, University of California Los Angeles, Los Angeles, California, United States of America; 6 Department of Molecular and Medical Genetics, Oregon Health and Science University, Portland, Oregon, United States of America; University of Washington, United States of America

## Abstract

Fibrillin-1 is a ubiquitous extracellular matrix molecule that sequesters latent growth factor complexes. A role for fibrillin-1 in specifying tissue microenvironments has not been elucidated, even though the concept that fibrillin-1 provides extracellular control of growth factor signaling is currently appreciated. Mutations in *FBN1* are mainly responsible for the Marfan syndrome (MFS), recognized by its pleiotropic clinical features including tall stature and arachnodactyly, aortic dilatation and dissection, and ectopia lentis. Each of the many different mutations in *FBN1* known to cause MFS must lead to similar clinical features through common mechanisms, proceeding principally through the activation of TGFβ signaling. Here we show that a novel *FBN1* mutation in a family with Weill-Marchesani syndrome (WMS) causes thick skin, short stature, and brachydactyly when replicated in mice. WMS mice confirm that this mutation does not cause MFS. The mutation deletes three domains in fibrillin-1, abolishing a binding site utilized by ADAMTSLIKE-2, -3, -6, and papilin. Our results place these ADAMTSLIKE proteins in a molecular pathway involving fibrillin-1 and ADAMTS-10. Investigations of microfibril ultrastructure in WMS humans and mice demonstrate that modulation of the fibrillin microfibril scaffold can influence local tissue microenvironments and link fibrillin-1 function to skin homeostasis and the regulation of dermal collagen production. Hence, pathogenetic mechanisms caused by dysregulated WMS microenvironments diverge from Marfan pathogenetic mechanisms, which lead to broad activation of TGFβ signaling in multiple tissues. We conclude that local tissue-specific microenvironments, affected in WMS, are maintained by a fibrillin-1 microfibril scaffold, modulated by ADAMTSLIKE proteins in concert with ADAMTS enzymes.

## Author Summary

The microenvironment is specified by cell-surface molecules, growth factors, and the extracellular matrix. Here we report genetic evidence that implicates fibrillin-1, a ubiquitous extracellular matrix molecule that sequesters latent growth factor complexes, as a key determinant in the local control of musculoskeletal and skin microenvironments. A novel mutation in fibrillin-1 demonstrates that modulation of the fibrillin microfibril scaffold can influence tissue microenvironments and result in the clinical features of Weill-Marchesani syndrome (WMS), including thick skin, short stature, and brachydactyly. Dysregulated WMS microenvironments diverge from Marfan pathogenetic mechanisms, which lead to broad activation of TGFβ signaling in multiple tissues.

## Introduction

Mutations in fibrillin-1 cause the pleiotropic features of the Marfan syndrome (MFS, OMIM#154700). MFS is recognized by its unique combination of skeletal, cardiovascular, and ocular features (long bone overgrowth, aortic root dilatation and dissection, and ectopia lentis). More than a thousand different mutations in *FBN1*, the gene for fibrillin-1, are known to cause MFS, suggesting that the same general pathogenetic mechanisms are initiated by each of these distinct mutations. In contrast, Weill-Marchesani syndrome (WMS, OMIM #608328) is a rare disorder described as “opposite” to MFS [1]. WMS, one of several types of acromelic chondrodysplasias, is characterized by short stature, brachydactyly, thick skin, and ectopia lentis. Previous studies reported that the autosomal dominant form of WMS is caused by mutations in *FBN1* [2], [3], while mutations in *ADAMTS10* were shown to cause recessive WMS [4], [5]. Since the clinical features of WMS and MFS may sometimes overlap [6], it is not certain how rare mutations in *FBN1* can bring about WMS instead of MFS. Additional investigations are required in order to clearly establish the role of fibrillin-1 in causing WMS.

A role for fibrillin-1 in skin fibrosis was first suggested when a mutation in *Fbn1* was identified in the tight-skin (tsk) mouse [7]. More recently, mutations in *FBN1* were found in Stiff Skin Syndrome (SSKS, OMIM #184900), a rare disorder characterized by hard, thick skin and joint contractures [8]. Both the tsk and SSKS phenotypes are caused by heterozygous mutations. However, the tsk mutation is a large in-frame gene duplication, while SSKS mutations are missense mutations confined to exon 37. The molecular mechanisms by which fibrillin-1 regulates skin fibrosis are obscure. Why the tsk and SSKS mutations do not cause MFS is also obscure.

Fibrillin-containing microfibrils are small diameter fibrils that are usually found in bundles or in association with elastic fibers. Individual fibrillin microfibrils are long and can be extended in vivo when tissues are under tension [9]. In humans and mice with MFS, fibrillin microfibril bundles were fragmented in the skin [10], [11]. In contrast, fibrillin microfibrils in human scleroderma skin were disorganized, when labeled and examined by immunoelectron microscopy [12]. The latter observation was extended by ultrastructural studies of fibrillin microfibrils in SSKS [8]. In SSKS, fibrillin microfibrils were found in large aggregates within which individual microfibrils appeared to be short [8]. These electron microscopic observations suggest that structural abnormalities in fibrillin microfibrils may underlie the differences between MFS and SSKS disease pathologies.

Another possibility is that mutations causing SSKS or WMS perturb growth factor signaling, since fibrillin-1 targets and sequesters the large latent Transforming Growth Factor β (TGFβ) complex [13], [14] as well as multiple Bone Morphogenetic Proteins (BMPs) and Growth and Differentiation Factor-5 (GDF-5) [15]–[17]. In MFS, abnormal activation of TGFβ signaling contributes to phenotypes in the lung [18] and aorta [19], but TGFβ signaling may not be abnormally activated in the skin. In SSKS or WMS skin, overproduction of collagen may be predicted to be due to abnormal activation of TGFβ. But, it is unclear why abnormal activation of TGFβ signaling would be limited to the skin in SSKS or WMS and alternatively to the lung and aorta in MFS. Different mechanisms may be involved in the activation of TGFβ signaling in these disorders and/or important unknown factors may limit the effects of mutations in fibrillin-1 to specific tissues.

Here we identify a novel mutation in fibrillin-1 in a family with WMS. In order to reveal the complex mechanisms by which fibrillin-1 differentially regulates connective tissues, we replicated this mutation causing human WMS in the mouse. We show that this mutation does not cause MFS, since the WMS mouse survives normally and does not display major features of MFS, even in homozygosity. Instead, WMS mutant mice develop skin fibrosis associated with distinctive ultrastructural abnormalities in fibrillin microfibrils. In addition, WMS mice demonstrate retardation of long bone growth. Therefore, WMS mice recapitulate cardinal features of human WMS. To elucidate molecular mechanisms of WMS, we provide evidence that the WMS mutation abolishes the binding site in fibrillin-1 for a novel family of proteins, the ADAMTSLIKE (ADAMTSL) proteins. We also connect ADAMTSL proteins with ADAMTS-10 and propose that these proteins form a complex with fibrillin-1. These findings implicate ADAMTSL proteins, together with ADAMTS-10, in the regulation of fibrillin microfibril structure. Insights gained from these studies are relevant to MFS and to the expanding genetic diseases that constitute the Marfan-related disorders. Furthermore, our results point out the importance of fibrillin-1 in the local regulation of tissue-specific microenvironments.

## Results

### Identification of an *FBN1* genomic deletion in a family with WMS

**Figure 1:**
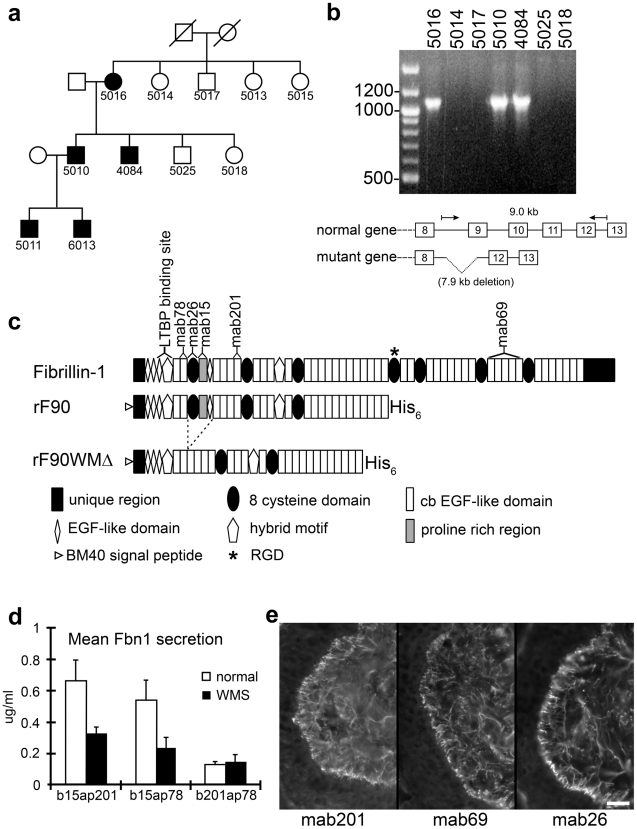
Characterization of a novel genomic deletion in *FBN1* in a family with WMS. (a) Partial pedigree of the family. Affected individuals are shown as filled symbols. (b) PCR from genomic DNA of selected family members, using primers flanking the deleted region. Only the affected individuals give rise to the shortened product (1.1 kb), which after DNA sequencing revealed a 7895 nt deletion with boundaries in *FBN1* introns 8 (IVS8-1207) and 11 (IVS11+1257). The wildtype product (9.0 kb) was too large to be amplified under the conditions used. (c) Schematic drawing of domains contained in the recombinant N-terminal half of wildtype (rF90) and WMS deleted fibrillin-1 protein (rF90WMΔ). The mutation results in the deletion of exons 9–11, encoding the first 8-cysteine domain, the adjacent proline rich region, and a generic EGF-like domain. Purity of preparations of rF90 and rF90WMΔ is shown in Figure S3. Epitopes of monoclonal antibodies used in this study are shown above the full-length molecule. In addition, the single RGD site and the binding site for LTBP are marked. (d) Sandwich ELISA used to quantitate secretion of wildtype and mutant fibrillin-1 in culture medium from normal dermal fibroblasts and WMS fibroblasts. Capture antibodies were biotinylated (b), and detector antibodies were coupled to alkaline phosphatase (ap). The capture and detector antibody pair that recognizes epitopes outside of the deleted region (b201 and ap78) detected both normal and WMS mutant fibrillin molecules and showed equal levels of fibrillin-1 secretion in both wildtype and WMS fibroblast medium. When mAb15 was used as a capture antibody, only wildtype fibrillin-1 was detected, because the proline-rich region, which contains the epitope recognized by mAb15 [33] is deleted in WMS fibrillin-1. Quantitation of fibrillin-1 secretion using the b15ap201 and b15ap78 antibody pairs showed approximately half the amount of fibrillin-1 in WMS fibroblast medium compared to medium from normal fibroblasts. These results indicate that equal amounts of normal and mutated fibrillin-1 are secreted by WMS fibroblasts. The differences in total fibrillin-1 amounts measured using the different pairs are due to differences in affinities of the pairs for the protein standard (rF11). For this experiment, n = 3 and the error bars represent the standard deviation. (e) Immunofluorescence of WMS skin showed a normal fibrillin pattern when mAb201, mAb69, or mab26 were used. Unlike immunofluorescence of Marfan skin [10], fibrillin-1 staining patterns were not fragmented in WMS skin. Scale bar = 20 µm.

A family with autosomal dominant WMS was previously described, and linkage analysis identified *FBN1* on chromosome 15q21.1 as the disease locus [2]. This family includes affected individuals in three generations (Figure 1a). Affected individuals exhibited characteristic features of WMS including microspherophakia, ectopia lentis, glaucoma, brachydactyly, short stature, and thickening of the skin, as previously documented for individuals 5016, 4084, and 5010 [2]. We further examined individuals 5010 (45 years of age), 5011 (15 years of age), and 6013 (10 years of age) and unaffected family members. Early removal of ocular lenses and short stature were common. Mild brachydactyly of the toe and some limitation of small joints were found in 6013. 5011 showed brachydactyly, more pronounced limitation of large and small joints, and thickened skin. 5010 showed severe limitation of small and large joints with pain and loss of dexterity and thick forearm skin without striae. In addition, all three individuals showed increased truncal and axial muscle bulk. No history or clinical evidence of valvular cardiac or aortic disease was found in this family.

Southern blotting of genomic DNA and PCR followed by DNA sequencing revealed a heterozygous 7895 nt genomic deletion in *FBN1* (Figure S1a and Figure 1b) with boundaries in introns 8 and 11. PCR results from unaffected and affected family members demonstrated that the mutation segregated with the disease (Figure 1b). Transcripts from the mutant allele lacked exons 9–11 (Figure 1b), predicting in-frame translation of fibrillin-1 molecules in which the first 8-cysteine domain, the proline-rich region, and EGF-like domain 4 are missing (Figure 1c). No similar mutations have been reported in the *FBN1* mutation database, where only 23 of 1,013 mutations were deletions or insertions [20]. By using ribonuclease protection assay, we were able to show that the wildtype and the mutant *FBN1* allele were equally expressed (Figure S1b). Using a quantitative sandwich ELISA, we found that wildtype and mutant fibrillin-1 proteins were equally secreted by affected WMS fibroblasts (Figure 1d). Immunofluorescence of skin from an affected individual (5011) showed fibrillin fibrils that appeared normal and not fragmented (Figure 1e), unlike the fragmented fibrils observed in MFS skin [10].

### Replication of the WMS mutation in mouse *Fbn1*

**Figure 2:**
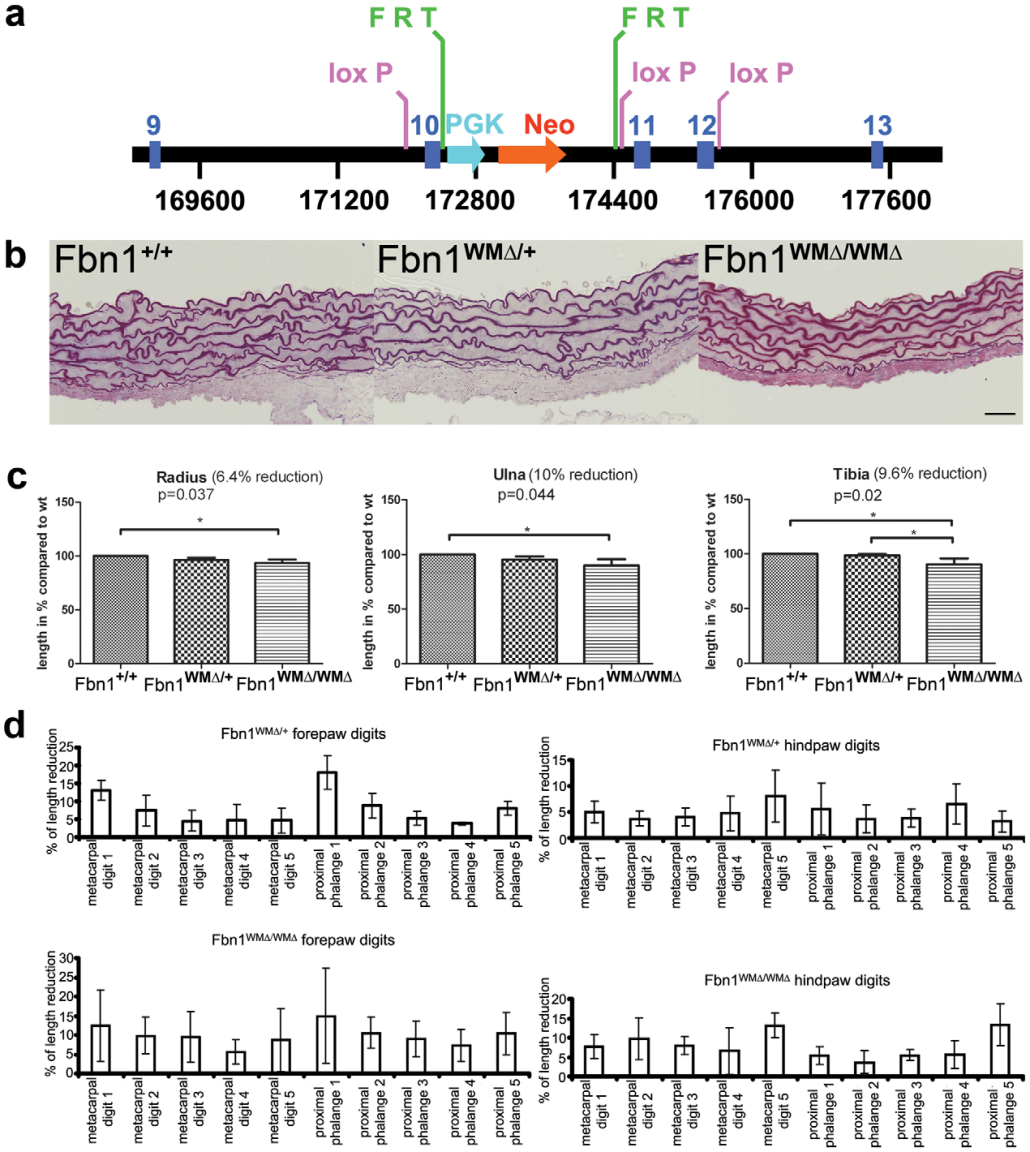
Replication of the WMS mutation in mice. (a) The targeted *Fbn1* locus. A neomycin selection cassette (PGK-Neo, flanked by FRT sites) was placed in the intron between exons 10 and 11. In addition, loxP sites were introduced before exons 10 and 11 and after exon 12. The neomycin cassette was removed by breeding targeted mice to FLPe mice. Cre-mediated recombination of the loxP sites resulted in deletion of exons 10–12, replicating the human WMS mutation. (b) Aortic root morphology. In contrast to Marfan mice, the aortic roots of 10 month old mutant mice showed no signs of fragmentation of the elastic lamellae. Scale bar = 25 µm. (c) Length measurements of long bones. μCT measurements revealed a reduction of 6–10% at 1 month of age, when homozygous mice were compared to gender-matched wildtype littermates (each bar represents the mean and standard deviation of measurements from 4 animals, n = 4). Significant p-values were obtained for all bones when comparisons were between homozygous and wildtype littermates. (d) Length measurements of skeletal elements in forepaws and hindpaws showed reduced digit length of metacarpals and proximal phalanges in WMΔ mutant mice relative to wildtype mice. All analyzed animals were gender matched littermates at 1 month of age (n = 5 for each genotype).

In contrast to the numerous different mutations in *FBN1* known to cause MFS, there is only one report of an *FBN1* mutation in a family with autosomal dominant WMS [3]. There are also reports of individuals with WMS that overlap with MFS [6]. In order to test whether the three-domain deletion in fibrillin-1 found in our family with WMS causes WMS and not MFS, we replicated the mutation in a mouse model (WMΔ) using a gene targeting strategy (Figure 2a). WMΔ heterozygous (WMΔ/+) and homozygous (WMΔ/WMΔ) mice breed well and are viable. Both heterozygous and homozygous mutant mice live longer than 1.5 years with no signs of aortic disease typical of MFS. Aortic root morphology in heterozygous and homozygous mutants is normal, even at 10 months of age (Figure 2b and Figure S2), in contrast to heterozygous and homozygous mutant mouse models of MFS [11], [19], [21]–[23]. In addition, with the exception of the mutant mgR/mgR [21], which is hypomorphic for normal *Fbn1* and dies during early adulthood, homozygous mutant mouse models of MFS die in the early postnatal period [11], [22], [23]. By these two major criteria for MFS in mice—aortic disease and early death of homozygotes—WMΔ mice do not model MFS.

Brachydactyly and short stature are features of WMS, while arachnodactyly and tall stature are characteristic of MFS. Therefore, long bones in the WMΔ mutant mice were measured. Growth of long bones appeared to be normal in the first two weeks of postnatal life but was reduced by 3–4 weeks of age in homozygous mice (WMΔ/WMΔ). Measurements of the long bones at 1 month of age in the WMΔ/WMΔ mice using μCT showed a statistically significant (P<0.05) reduction in lengths of the radius, ulna, and tibia of 6–10% (Figure 2c) compared to age and gender matched wildtype controls. At 3–4 weeks of age, length measurements of metacarpals and proximal and distal phalanges in fore- and hindpaws were also reduced between 2–23% in the WMΔ/+ and WMΔ/WMΔ mice (Figure 2d). These findings are consistent with the WMS phenotype. However, by 5 months of age, these differences in length were normalized.

### Skin fibrosis in WMΔ mutant mice

**Figure 3:**
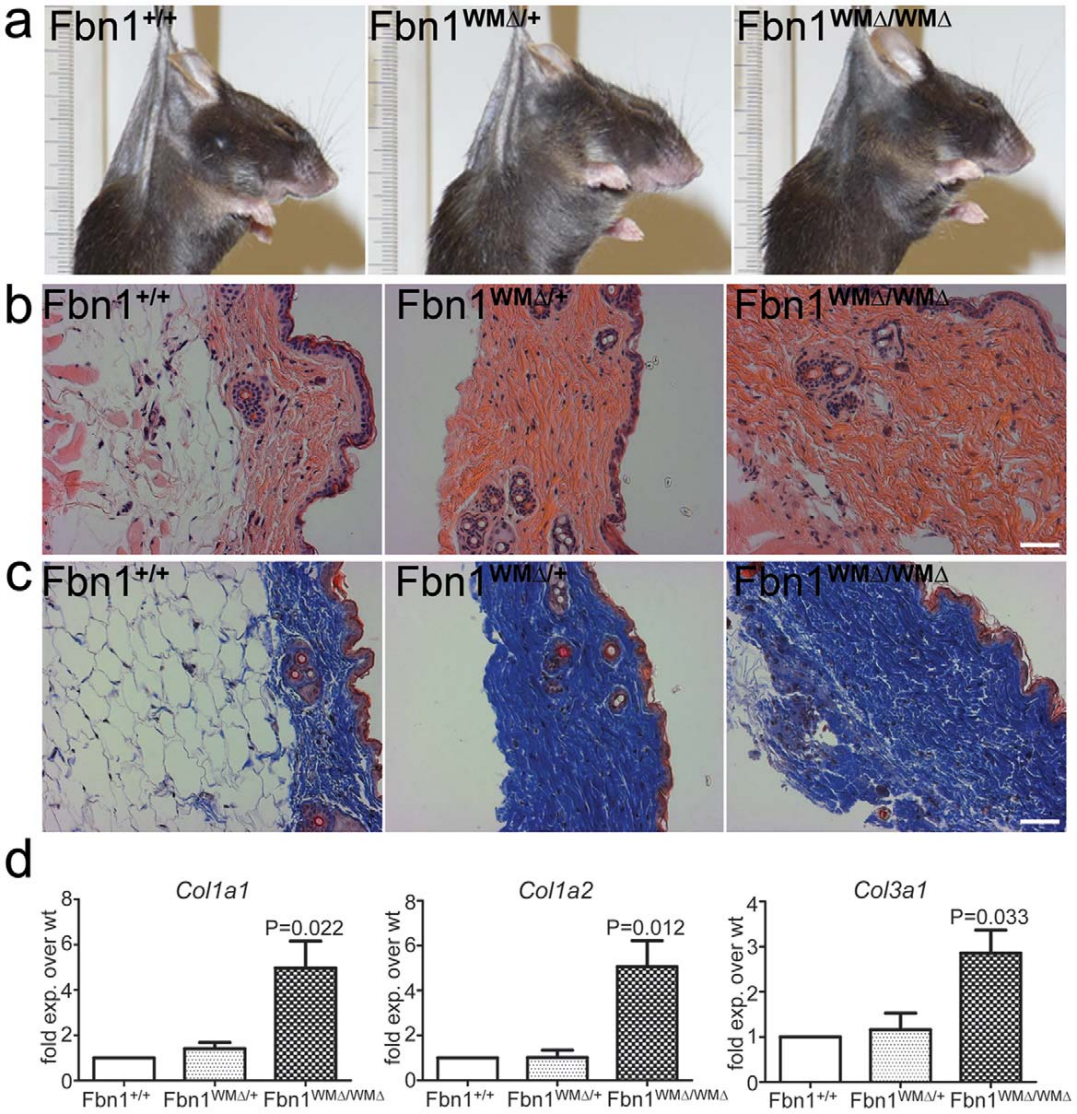
Thick skin phenotype in WMΔ mice. (a) Gross inspection. 7.5 month WMΔ mice could be identified by touch. Skin felt thicker and was less elastic in both heterozygous (WMΔ/+) and homozygous (WMΔ/WMΔ) mice compared to wildtype (Fbn1^+/+^) littermates. Here, mice were sacrificed, shaved, and immediately suspended by forceps positioned at the same relative spot between the ears. The ruler is included to show equivalent magnifications in the photographs. (b) Hematoxylin and eosin staining of skin from 10 month old WMΔ littermates. Dermal fibers in the mutant skin appeared to be thicker and more densely packed when compared to the wildtype littermate skin. Scale bar = 50 µm. (c) Masson's trichrome staining of skin from the same 10 month old littermates as in (b). WMΔ heterozygous and homozygous mutant skin showed increased collagen deposition. In addition, in both (b) and (c), the dermis appeared to be wider with a strikingly diminished hypodermal fat layer. Scale bar = 50 µm. (d) qPCR of collagen genes. RNAs were extracted from skin from WMΔ wildtype and mutant littermates (n = 5 for each genotype), and collagen genes were quantitated by qPCR. Type I and Type III collagen gene expression was found to be significantly upregulated in the homozygotes.

Gross examination suggested a thickened, less elastic skin in WMΔ mutant mice (Figure 3a). Histology of skin biopsies from WMΔ mice showed excessive collagen deposition in the dermis starting at 1 month of age. Hematoxylin and eosin or Masson's Trichrome stains (Figure 3b, 3c) revealed a widened dermal layer with decreased hypodermal fat, and thicker, more densely packed collagen fibers in mutants compared to wildtype mice. qPCR analyses demonstrated upregulated expression of collagen genes in the skin from mutant mice (Figure 3d). Skin thickness, as determined by histological stains and detected by touch by 7 months of age, persisted through old age in the mutant mice.

**Figure 4:**
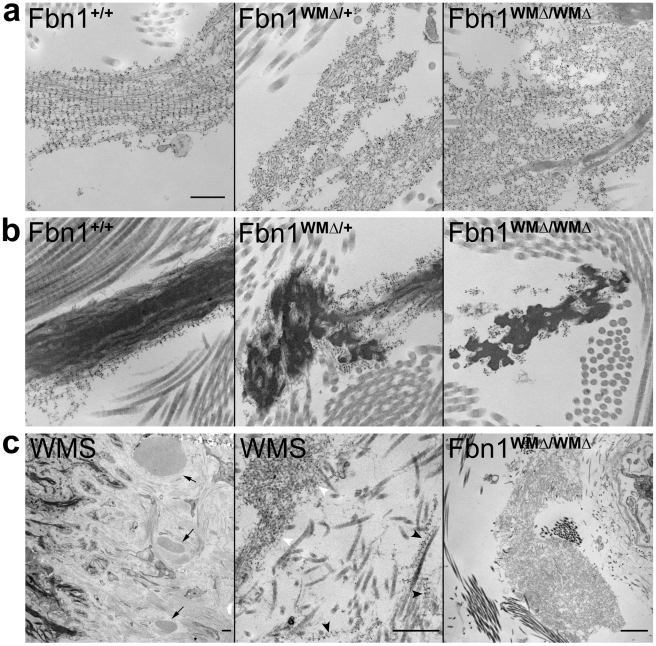
Ultrastructural abnormalities in microfibrils in WMΔ mouse and WMS human skin. (a,b) Immunogold labeling of 9 month old wildtype and WMΔ mutant skin. Wildtype microfibrils decorated with fibrillin-1 antibodies showed periodic gold labeling along the lengths of individual microfibrils in the absence of elastin (Fbn1^+/+^, a) and on the periphery of amorphous, darkly stained elastin cores (Fbn1^+/+^, b). In contrast, fibrillin microfibrils in WMΔ mutant skin showed reduced immunogold periodicity along microfibrils and larger and denser accumulations of microfibril aggregates (WMΔ/+ and WMΔ/WMΔ, a); elastic fibers appeared to be moth-eaten and also showed reduced fibrillin-1 periodic labeling (b). Scale bar = 300 nm for all panels in a,b. (c) Extreme ultrastructural appearance of abnormal microfibril aggregates in both human WMS and mouse WMΔ skin. Large dense aggregates of microfibrils were easily identified at low magnification in the skin of an 18 year old individual with WMS (left panel, arrows). Immunogold labeling demonstrated both normal periodic microfibrils (black arrowheads) and irregularly labeled microfibrils (white arrowheads) (middle panel). Large dense aggregates of microfibrils were also found in older WMΔ mutant mouse skin. One such aggregate is shown in skin from a 17 month old homozygous (WMΔ/WMΔ) mouse. Scale bars = 500 nm.

Electron microscopy after immunogold labeling with anti-fibrillin-1 antibodies showed alterations in fibrillin microfibril ultrastructure in WMΔ/+ and WMΔ/WMΔ skin: large bundles of microfibrils as well as microfibrils around elastin cores showed reduced periodicity of immunogold labeling in the mutants (Figure 4a,4b). In addition, large accumulations of microfibrils were prominent in WMΔ/+ and WMΔ/WMΔ skin (Figure 4a), and elastic fibers appeared moth-eaten compared with wildtype littermates (Figure 4b). The disorganized appearance of microfibrils is better visualized in the three-dimensional aligned tilt series of immunolabeled microfibrils from WMΔ/WMΔ and wildtype skin samples supplied Videos S1 and S2.

While some areas of the skin appeared normal, electron microscopic examination of skin from an 18 year old individual with WMS (unrelated to the WMS family described above) revealed unusually large abnormal aggregates of microfibrils (visible at low magnification in Figure 4c, left panel, arrows). Elastic fibers also appeared moth-eaten (data not shown). Similar to observations of WMΔ mutant mouse skin (Figure 4a), immunogold labeling with antibodies specific for fibrillin-1 demonstrated both irregular labeling of the microfibril aggregates and periodic labeling of apparently normal microfibrils (Figure 4c, middle panel). Based on these immunolocalization results with antibodies specific for fibrillin-1, we conclude that the small and large aggregates are composed of abnormal bundles of fibrillin-1 microfibrils. Large microfibril aggregates similar to those in human WMS (Figure 4c, left and middle panels) were found in skin from older (11–20 month old) WMΔ/WMΔ mice (Figure 4c, right panel). These results provide evidence for a common pathogenetic mechanism for fibrosis in human WMS and in this mouse model of WMS.

### Uncovering a new molecular pathway for WMS

**Figure 5:**
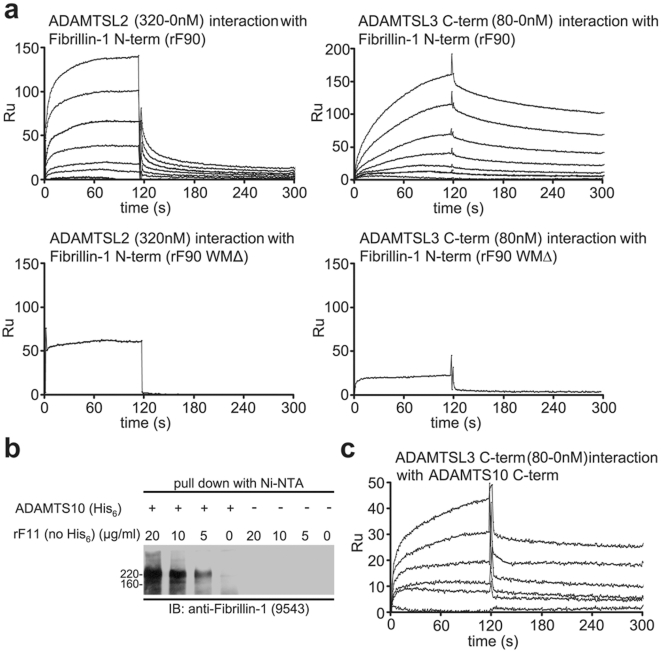
Biochemical analyses of interactions among ADAMTSL proteins, fibrillin-1, and ADAMTS-10. (a) SPR sensorgrams showing binding of different concentrations of soluble ligands to the N-terminal half of fibrillin-1 (rF90), coupled onto a chip. Full-length ADAMTSL-2 (320-0 nM) interacts with rF90, as does the C-terminal end of ADAMTSL-3 (80-0 nM). No binding was detected when rF90WMΔ was used, demonstrating that the binding site for ADAMTSL proteins resides in the deleted region. (b) Fibrillin-1/ADAMTS-10 pull-down assay. Conditioned medium from transfected cells expressing full-length ADAMTS-10 with a C-terminal His_6_-tag was incubated with increasing amounts of rF11 (N-terminal half of fibrillin-1, similar to rF90 but lacking the His_6_-tag). ADAMTS-10 complexes were pulled down after incubation with Nickel NTA resin. SDS-PAGE followed by immunoblotting (IB) with anti-fibrillin-1 antibody (9543) showed the presence of fibrillin-1 in the pulled-down ADAMTS-10 complexes. Conditioned medium from untransfected cells (−) was subjected to the same procedure and served as a control. Even though rF11 was added in similar amounts to the control medium, no fibrillin-1 was pulled-down, demonstrating the specificity of the interaction between ADAMTS-10 and fibrillin-1. (c) SPR sensorgrams showing binding of different concentrations (80-0 nM) of soluble C-terminal ADAMTSL-3 to the C-terminal end of ADAMTS-10, coupled to a chip. Calculated K_D_ for this interaction was 2 nM.

We previously showed that ADAMTSL-6 interacts with the N-terminal half of fibrillin-1 with high affinity (K_D_ = 80 nM) [24]. In order to test whether other ADAMTSL family members also bind to fibrillin-1 and whether the binding site utilized by ADAMTSL-6 is perturbed by the WMS three-domain deletion in fibrillin-1, we generated recombinant fibrillin-1 polypeptides, rF84 and rF84WMΔ (Figure S3) and rF90 and rF90WMΔ (Figure 1c), as well as recombinant human ADAMTSL-1, -2, -3, and mouse papilin polypeptides (Figure S3). Surface plasmon resonance (SPR) technology was employed to measure interactions between fibrillin-1 and ADAMTSL polypeptides. Similar to ADAMTSL-6 [24], ADAMTSL-2, -3, and papilin polypeptides interacted with the N-terminal half of fibrillin-1, while ADAMTSL-1 did not. Binding to the C-terminal half of fibrillin-1 was negative for all ADAMTSL proteins tested (data not shown). SPR sensorgrams are shown for ADAMTSL-2 binding to fibrillin-1 and for ADAMTSL-3 binding to fibrillin-1 (Figure 5a). ADAMTSL-2, -3, and -6 and papilin polypeptides did not bind to recombinant fibrillin-1 polypeptides with the WMS three-domain deletion (Figure 5a and Table S1). Binding constants for all of these interactions were calculated from the SPR data (Table S1). We conclude that the fibrillin-1 domains consisting of the first 8-cysteine domain, the proline-rich region, and the 4^th^ generic EGF-like domain contain the ADAMTSL binding site(s).

Because recessive WMS is caused by mutations in *ADAMTS10* [4], [5], *FBN1* and *ADAMTS10* share a genetic pathway. Therefore, we hypothesized that fibrillin-1, ADAMTS-10, and some ADAMTSL proteins form protein complexes. In a pull-down assay with Ni-NTA as a resin, we showed that full-length, His_6_-tagged ADAMTS-10 in media of stably transfected EBNA 293 cells bound to the N-terminal half of fibrillin-1 (Figure 5b). From SPR interaction studies, a K_D_ of 450 nM was calculated for the binding of the N-terminal fibrillin-1 polypeptide to the C-terminal end of ADAMTS-10 (data not shown). The C-terminal recombinant ADAMTS-10 polypeptide used in the SPR studies represents the noncatalytic region of ADAMTS-10, a region composed primarily of Tsp1 repeats (Figure S3). SPR also showed that the C-terminal end of ADAMTS-10 interacted with the C-terminal end of ADAMTSL-3 with high binding affinity (K_D_ = 2 nM) (Figure 5c). However, neither ADAMTSL-2 nor -1 bound to ADAMTS-10, indicating that ADAMTS enzymes may partner only with specific ADAMTSL proteins. Taken all together, these results suggest that direct interactions between fibrillin-1, ADAMTS-10, and specific ADAMTSL proteins are involved in the pathogenesis of WMS.

### Mechanisms contributing to pathogenesis of WMS

**Figure 6:**
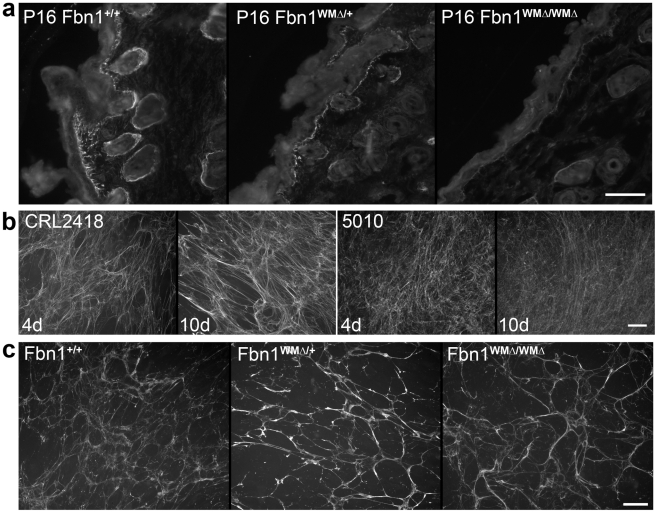
Immunofluorescence analyses of WMS skin and fibroblast cultures. (a) Immunolocalization of ADAMTSL-6 on P16 wildtype, heterozygous and homozygous WMΔ littermates. Antibodies specific for ADAMTSL-6 [24] were used to stain littermate skin. As previously shown [24], ADAMTSL-6 staining was similar to fibrillin-1 immunostaining in wildtype skin. WMΔ mutant skin showed a reduction in ADAMTSL-6 immunostaining, indicating that deletion of the ADAMTSL binding site in fibrillin-1 prevents ADAMTSL-6 from binding to fibrillin-1 in vivo. (b) Human fibroblast cell cultures stained with antibodies to fibrillin-1 (pAb9543) after 4 days and 10 days of culture. Control (CRL2418) fibroblasts elaborated a typical fibrillin-1 fibril matrix that was clearly abundant at day 4 and day 10. In contrast, WMS (5010) fibroblasts deposited abundant fibrillin-1 fibrils at day 4, but these fibrils were thinner and more diffuse. Even after 10 days in culture, the WMS fibroblasts failed to establish prominent bundles of fibrillin fibrils. (c) Fibroblast cultures from wildtype, heterozygous, and homozygous WMΔ mice stained with anti-fibrillin-1 (pAb9543) after 4 days in culture. Mutant fibroblast cultures elaborated typical abundant, long fibrillin-1 fibrils that appeared somewhat thicker than fibrils in wildtype cultures. Scale bars = 50 µm.

Based on in vitro studies, we hypothesized that the mutant WMS fibrillin-1 cannot interact properly in vivo with certain members of the ADAMTSL family of proteins. To test if localization of ADAMTSL proteins is altered in WMΔ mutant mice, we stained skin with antibodies specific for ADAMTSL-6 [24]. Results showed a reduction in ADAMTSL-6 immunofluorescence in skin from WMΔ/+ and WMΔ/WMΔ mutant mice compared to wildtype littermate skin (Figure 6a). Fibrillin-1 immunofluorescence was equal in pattern and abundance in WMΔ mutant and wildtype mice (data not shown). Since antibodies specific for ADAMTSL-2 and -3 are not yet available, we were unable to determine whether these proteins also colocalize with fibrillin-1 in skin and whether these are also reduced in WMΔ mutant mice.

ADAMTSL-6 and ADAMTS-10 promote fibrillin-1 fibril formation [24], [25]. Therefore, we examined human and mouse fibroblasts for defects in fibrillin-1 fibril formation. The Marfan cell culture assay [10] was used. In this assay, control fibroblasts assemble a matrix containing abundant immunofluorescent fibrillin-1 fibrils, while MFS fibroblasts assemble only few or no fibrils [10]. Unlike MFS fibroblasts, fibroblasts from a member (5010) of the WMS family described here showed abundant immunofluorescent fibrillin-1 fibrils (Figure 6b). However, these fibrils appeared to be much thinner and less bundled than control (CRL2418) fibrillin-1 fibrils. Fibroblasts from wildtype, heterozygous and homozygous WMΔ littermates also showed abundant immunofluorescent fibrillin-1 fibrils (Figure 6c). Immunofluorescence staining of WMΔ/+ and WMΔ/WMΔ cultures suggested thicker bundles of fibrillin-1 fibrils than those in wildtype cultures (Figure 6c). These results underscore the conclusion that the WMS mutation in fibrillin-1 works mechanistically differently than other *FBN1* mutations that cause MFS. In addition, results suggest that the mutant WMS fibrillin-1 causes defects in fibrillin-1 fibril aggregation or bundling, but observed differences between the human WMS and the mouse WMΔ cultures cannot currently be explained.

**Figure 7:**
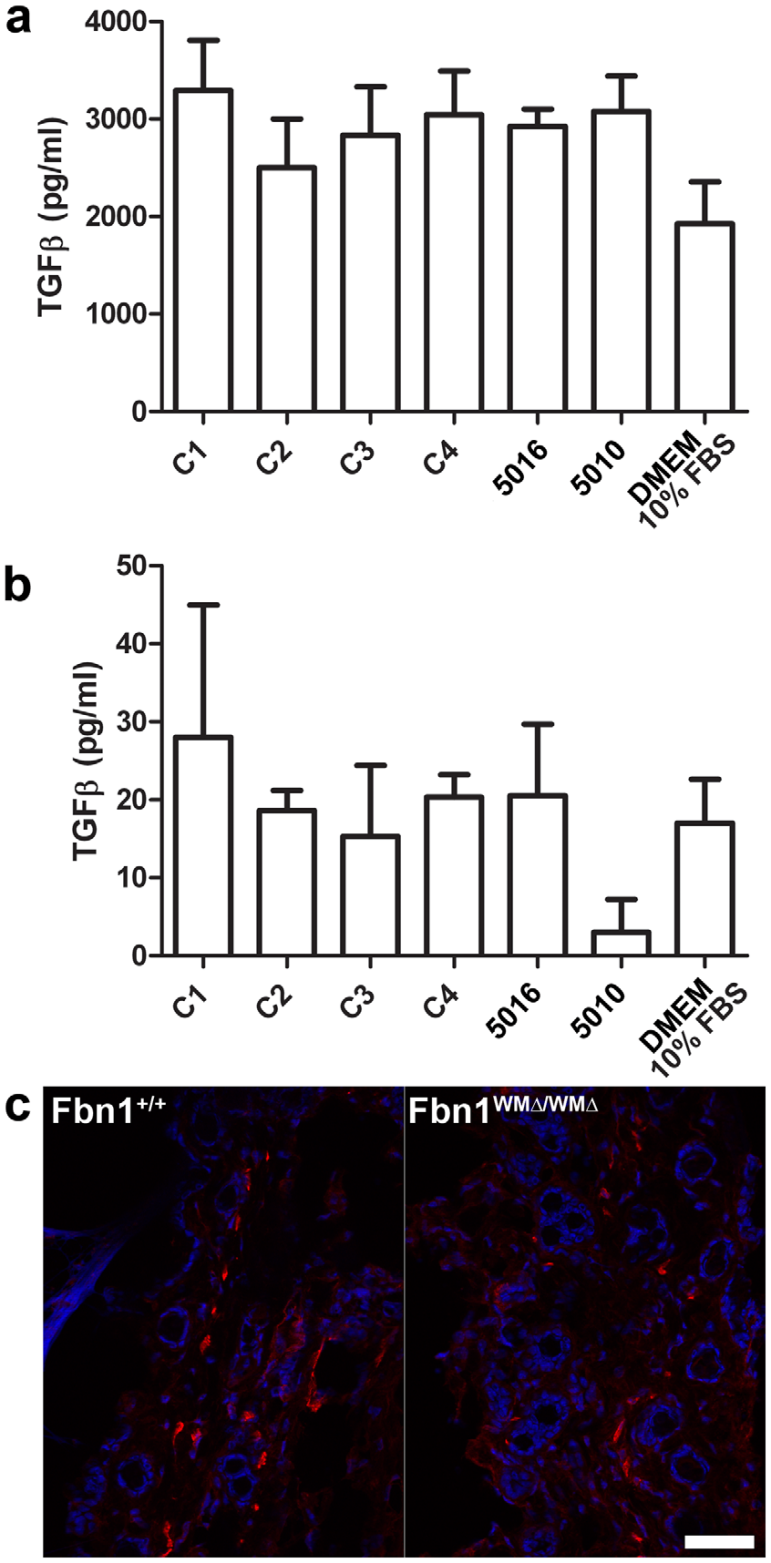
Measurements of TGF-β in cultured fibroblasts; α-smooth muscle actin staining. (a) WMS fibroblasts (family members 5016 and 5010) secreted equal amounts of total TGF-β protein compared to controls (C1, C2, C3, and C4). (b) No significant differences were detected in amounts of active TGF-β present in the media of WMS fibroblasts compared to controls. Medium containing 10% fetal bovine serum was used to show baseline values. For experiments in (a) and (b), n = 2 or 3, and the error bars represent the standard deviation. (c) Skin from 6-month old wildtype and WMΔ/WMΔ littermates showed no difference in numbers of cells stained by an α-smooth muscle actin antibody (red). DAPI nuclear stain is blue. Scale bar = 20 µm.

Another potential mechanism contributing to pathogenesis of WMS is abnormal activation of TGFβ signaling. Our findings of upregulated collagen genes in the skin of WMΔ mice and increased Trichrome staining of WMΔ dermis (Figure 3) suggested increased TGFβ signaling. However, Western blotting for pSmad 2/3 showed no differences between wildtype and WMΔ skin at multiple time points (data not shown), and qPCR quantitation of TGFβ-responsive genes such as *Ctgf*, *Pai1*, and *Postn* also demonstrated no differences at multiple time points (data not shown). When total (Figure 7a) and active TGFβ (Figure 7b) were measured in the medium of human cultured fibroblasts, no significant difference was found between control and WMS fibroblasts. Skin samples from heterozygous and homozygous WMΔ mice of different ages were examined for the presence of myofibroblasts. Staining with antibodies specific for α-smooth muscle actin did not reveal increased numbers of myofibroblasts in mutant WMΔ mice (Figure 7c).

We also tested interactions between Latent TGFβ Binding Proteins (LTBPs) and ADAMTSL proteins, since an interaction between ADAMTSL-2 and the middle region of LTBP-1 was previously identified [26]. SPR binding studies (summarized in Table S2) showed no interaction between ADAMTSL-2 and the middle region of LTBP-1 (rL1-M [13]). However, binding between ADAMTSL-2 and the C-terminal domains of LTBP-1 present in rL1-K [13] was detected. Binding between ADAMTSL-3 and the C-terminal domains of LTBP-1 was also detected, but neither ADAMTSL-2 nor -3 interacted with LTBP-4.

## Discussion

WMS is considered to be clinically homogenous, even though the genetic basis of WMS is heterogeneous [27]. Both recessive and dominant forms of WMS present equally with myopia, glaucoma, cataract, short stature, brachydactyly, thick skin, and muscular build. There may be significant differences in incidence of microspherophakia, ectopia lentis and joint limitations between the recessive and dominant forms [27], but these features are also common to both. Currently, there is a single report of a mutation in *FBN1* in a family with dominant WMS [3].

Results presented here identify a novel mutation in *FBN1* in a family with dominant WMS, which was previously linked to *FBN1* [2]. The mutation is predicted to result in fibrillin-1 molecules that lack the first 8-cysteine domain, the proline-rich region, and the adjacent EGF-like domain. Replication of this mutation in mouse *Fbn1* clearly demonstrated that the mutation reproduces at least one cardinal feature of WMS—thick skin—and does not cause the clinical equivalent of MFS. Reduced long bone growth was also found in WMΔ mice, consistent with the human WMS traits of short stature and brachydactyly. However, by 5 months of age, long bone growth was normalized, perhaps reflecting differences between humans, in whom growth plate closure occurs at skeletal maturity, and rodents, whose growth plates fail to close [28], and who grow for a longer period of time than humans using cellular processes which are not active in adult humans [29]. In addition, hypermuscularity, a feature of human WMS, is also found in WMΔ mutant mice (data not shown).

**Figure 8:**
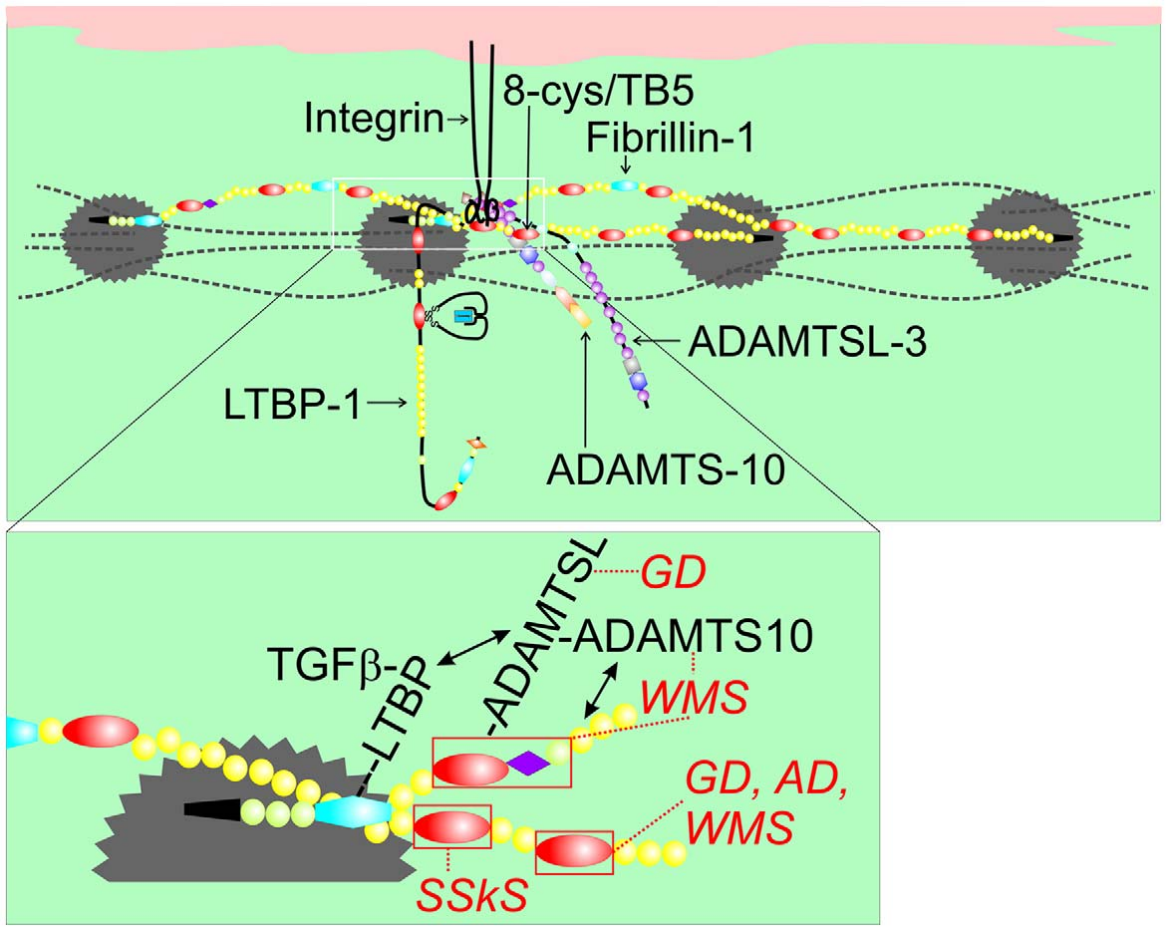
Model of fibrillin-1 containing microfibrils showing the locations of binding sites for ADAMTSL proteins, LTBP-1, and integrins. This model of fibrillin-1 molecules arranged as parallel, staggered molecules within the beads-on-a-string microfibril was previously proposed [33]. Two staggered fibrillin-1 molecules are shown with colored domains (see Figure 1c for domain structure), while other fibrillin molecules within the microfibril are depicted as dashed black lines. Beaded regions of the microfibril are represented as gray scalloped circles. The inset shows the N-terminus (black) of one molecule extending through cbEGF5 and crossing over the middle portion of a second molecule (shown from Hybrid2 through cbEGF27). In this model, binding sites for ADAMTSL proteins (within the first 8-cysteine domain, the proline-rich domain, and the adjacent generic EGF-like domain) and for LTBP-1 (within the first hybrid domain) on one molecule are very close to the integrin-binding RGD site (contained in the fourth 8-cysteine domain) on a second molecule. Mutations in the fourth 8-cysteine domain can cause SSKS, presumably by disrupting integrin binding. The fifth 8-cysteine domain or TB5 contains mutations in *FBN1* that result in WMS [3], geleophysic (GD) or acromicric dysplasia (AD) [34]. Mutations in *ADAMTSL2* also lead to GD, and mutations in *ADAMTS10* lead to WMS. We propose that this cluster of molecular interactions (magnified in the inset) constitutes a microenvironment controlling thick skin and musculoskeletal growth.

Biochemical studies comparing wildtype and WMS mutant fibrillin-1 revealed that the WMS mutation abolished a specific binding site in fibrillin-1 for ADAMTSL-2, -3, -6 and papilin. Further biochemical studies suggested that specific ADAMTSL proteins may interact with ADAMTS-10 and that ADAMTS-10 binds to fibrillin-1. Our results are consistent with previous studies of papilin, the first of the ADAMTSL proteins to be described, and procollagen N-proteinase (now called ADAMTS-2), which indicated that the “papilin cassette” (homologous to the noncatalytic regions of ADAMTS enzymes) may interact with ADAMTS metalloproteinases [30]. In addition, binding between ADAMTS-10 and fibrillin-1 was recently demonstrated [25]. Therefore, we propose that a ternary complex of ADAMTSL, ADAMTS-10, and fibrillin-1 may be formed. Such a ternary complex is depicted in Figure 8, showing how ADAMTSL-3 and ADAMTS-10 may bind as a complex to fibrillin-1 in microfibrils.

Mutations in *ADAMTSL2* cause geleophysic dysplasia [26]. Mutations in *ADAMTS17* cause a Weill-Marchesani-like syndrome [31], and mutations in *ADAMTSL4* cause autosomal recessive isolated ectopia lentis [32]. Both geleophysic dysplasia and WMS are acromelic dysplasias sharing features of short stature, brachydactyly, thick skin, limited joint mobility, and hypermuscularity. Ectopia lentis is a common feature of MFS and WMS. All together, these related genetic disorders suggest that specific ADAMTSL (at least ADAMTSL-2 and -4) and ADAMTS (ADAMTS-10 and -17) proteins modulate fibrillin-1 function in the skeleton, skin, joints, muscle, and eye. Our biochemical data also implicate ADAMTSL-3 and -6 in these pathways. Whether all members of the ADAMTSL/ADAMTS family perform similar roles in the modulation of fibrillin-1 function is unknown. However, if similar functions are performed, differences in temporal and spatial regulation of the expression of these genes could account for tissue-specific variation in these related disorders.

An in-frame deletion of 24 nucleotides was found in *FBN1* to cause autosomal dominant WMS [3]. This mutation (5074_5097del) deletes 8 amino acid residues (R^1692^ – Y^1699^) from the fifth 8-cysteine domain (also called TB5) in fibrillin-1. When fibrillin-1 is modeled within microfibrils [33], the fifth 8-cysteine domain in one molecule of fibrillin-1 is close to the ADAMTSL binding site in an adjacent fibrillin-1 molecule (Figure 8). Recently, 16 novel heterozygous mutations in *FBN1* causing geleophysic dysplasia or acromicric dysplasia were also identified in the fifth 8-cysteine domain [34]. WMS, geleophysic dysplasia, and acromicric dysplasia are members of the acromelic group of dysplasias with similar as well as distinctive clinical features. In our model, the clustering of fibrillin-1 domains associated with acromelic dysplasias and with binding sites for ADAMTSL proteins involved in acromelic dysplasias may specify a new microenvironment controlling thick skin and musculoskeletal growth.

It is interesting that, when fibrillin-1 is modeled within microfibrils [33], the single RGD-containing domain in fibrillin-1 is close to the ADAMTSL binding site in fibrillin-1 (Figure 8). Integrin binding to RGD sites is known to perform important roles in matrix assembly [35] and to be critically dependent on the surrounding sequences, which can silence RGD function [36]. SSKS is caused by missense mutations in *FBN1* exon 37, which encodes the domain containing the RGD site [8]. Therefore, it can be speculated that SSKS mutations in *FBN1* lead to diminished integrin activity. Because dermal fibrosis and the abnormal ultrastructural appearance of fibrillin microfibrils were similar in both SSKS [8] and WMS (Figure 4), it seems likely that integrin interactions with fibrillin-1 are perturbed in both SSKS and WMS. Furthermore, the proximity of the RGD-containing domain to the ADAMTSL binding site in fibrillin-1 suggests that integrins may cooperate with ADAMTSL proteins and ADAMTS-10 in modulating fibrillin microfibril [24], [25] aggregation and organization. However, the molecular mechanisms of this cooperation remain unknown.

Abnormal TGFβ signaling may play a role in these disorders, since fibrillin-1 microfibrils target and sequester the large latent TGFβ complex [13]. We found upregulated collagen gene expression and increased Trichrome staining in the skin of WMΔ mutant mice, results that are consistent with activated TGFβ signaling. In addition, molecular interactions of LTBP-1 with both ADAMTSL-2 and ADAMTSL-3 were determined, suggesting that loss of the ADAMTSL binding site in WMΔ mutant mice might render the large latent TGFβ complex more susceptible to activation. However, if activation of TGFβ signaling underlies the dermal fibrosis in WMΔ mutant mice, this activation of signaling did not manifest detectable differences in other conventional TGFβ signaling readouts (e.g., increased α-smooth muscle actin positive cells). It has been recently speculated that mechanical forces may be required to activate the latent TGFβ complex [37]. Therefore, it is possible that local changes in the fibrillin microfibril matrix could influence force-dependent activation of latent TGFβ, perhaps leading to a local increase in signaling.

Activation of TGFβ signaling has been shown for geleophysic dysplasia [26], [34], acromicric dysplasia [34], and for MFS [18], [19]. We propose that, in consort with the different tissue-specific manifestations of disease in WMS and MFS, activation of TGFβ signaling in these diseases may be limited (WMS) or more global (MFS) in scope. In MFS, the broad activation of TGFβ signaling in multiple tissues matches the pleiotropic features of the disease and the requirement for general pathogenetic mechanisms initiated by multiple different disease-causing mutations. In the case of WMS, we propose that fibrosis limited to the skin is due to the dysregulated interaction between abnormally organized microfibrils and the large latent TGFβ complex within dermal microenvironments. Our data suggest that direct interactions between ADAMTSL proteins, fibrillin-1, and LTBP-1 (Figure 8) may be dysregulated in WMS, leading to concomitant structural and signaling abnormalities within local spaces. However, the discrepancy in the data measuring TGFβ activity between our WMS fibroblast cultures and geleophysic and acromicric dysplasia fibroblast cultures [26], [34] is not yet understood. Although further investigations are required in order to determine the roles of ADAMTSL/ADAMTS-10 complexes, integrins, and LTBP-1 in the fine regulation of TGFβ signaling in WMS skin fibrosis, we conclude that these molecular pathways work locally—in microenvironments—to control skin fibrosis. While the importance of the microenvironment is appreciated in development and cancer [38], this is to our knowledge the first evidence for microenvironmental regulation by fibrillin-1.

In summary, our results suggest an improved concept of the architectural and regulatory functions of fibrillin-1. Previously, the microfibrils of elastic, distensible tissues were thought to function mechanically only as a limiting component for a cross-linked, isotropic elastin matrix. Subsequently, the attachment of LTBPs and BMPs demonstrated that fibrillin microfibrils participate in the storage and release of growth factors. Now we show that fibrillin-1 also selectively binds the metalloproteinase ADAMTS-10 and some non-enzymatic ADAMTSL proteins, enabling a clustering of these protein complexes in the vicinity of the fibrillin-1 RGD site and suggesting the potential for integrin involvement in ADAMTS/ADAMTSL/fibrillin functions. The established mutual affinity of the protein components of this cluster opens varied biochemical pathways that need to be explored in the future. The genetic evidence in humans and mice shows that perturbation of such biochemical pathways can lead to significant pathobiological consequences. In addition, the genetic evidence clearly demonstrates that fibrillin-1 microfibrils, although ubiquitous structural elements in the connective tissue space, perform local functions to support tissue microenvironments.

From the perspective of normal development and tissue homeostasis, we propose that the fibrillin microenvironment may enable two-way communication between a cell and its surroundings. The extended fibrillin fibril may function as a sensor for mechanical distortion of the matrix, signaling the cell when there is a need for additional, reinforcing structural components like collagens. The presence of large latent TGFβ complexes within the fibrillin microenvironment conveniently couples matrix mechanics with available signals for upregulation of collagens. Installation of new structural materials into pre-existing matrices likely requires remodeling enzymes like metalloproteinases. ADAMTS enzymes, localized to the fibrillin microenvironment as well, possibly with the help of ADAMTSL proteins, could serve such purposes, or they might participate in the activation of nearby latent growth factors. Understanding pathogenetic mechanisms underlying WMS and MFS will elucidate the local, fine adjustments required for growth, homeostasis, and repair.

## Materials and Methods

### Ethics statement

Clinical studies were performed with informed consent and local OHSU Internal Review Board approval. All mouse work was approved by the OHSU IACUC committee.

### Individuals with WMS

The family pedigree shown in Figure 1 has been previously described [2]. Dermal fibroblast cultures were established from punch biopsies obtained with informed consent and local OHSU IRB approval. All participants were evaluated for myopia, glaucoma and dislocated lenses, for musculoskeletal and skin characteristics, and for cardiac or aortic disease assessed by history and/or by auscultation. This family has been followed for more than 15 years with no clinical evidence of valvular cardiac or aortic disease. A second unrelated individual, designated WMS2, was seen who had been previously diagnosed with WMS. At 18 years old, she had a history of early high myopia and presented with headaches secondary to glaucoma. She had short stature, mild brachydactyly, microspherophakia, and apparently normal joints and skin. A punch biopsy was obtained with informed consent.

### Mutation detection

Genomic DNA was extracted from cultured WMS 5016 or normal skin fibroblasts (NSF) or EDTA whole blood using standard procedures. Individual *FBN1* exons were amplified by PCR of genomic DNA using intronic primers. Overlapping *FBN1* cDNAs were amplified by PCR using exonic primers (for sequences see Table S3). PCR products were sequenced.

### Southern blotting

Genomic DNA was digested (using HindIII, Bsu36I, NcoI, SpeI, SspI), separated electrophoretically and transferred to a nylon membrane. The membrane was probed with a 727 bp cDNA fragment of *FBN1* encompassing exons 8–12, radiolabeled with ^32^P-αdCTP in the presence of random and specific primers, and exposed after stringent washing to film for autoradiography.

### Ribonuclease protection assay

Primers flanking *FBN1* exons 9, 11, 21, and 37 were used to generate fragments of genomic NSF DNA, and the products were cloned into the pGEM-T-Easy vector (Promega). Radiolabeled probes were hybridized to total RNA of normal or WMS patient fibroblasts, followed by ribonuclease digestion to degrade unhybridized regions (RPA III kit, Ambion). Protected fragments were separated by acrylamide gel electrophoresis, and quantitated by phosphorimager (STORM, Molecular Diagnostics).

### Generation of mice

All materials used for the generation of the WMΔ mouse line originated from C57BL/6 mice (see Figure 4 for design of targeting vector). The floxed WMΔ mouse line was generated by Ozgene Pty. Ltd. (Bentley, Australia). The neomycin selection cassette was removed by breeding targeted mice to FLPe mice. Cre-mediated removal of *Fbn1* exons 10–12 in all cells was accomplished by breeding floxed WMΔ mice to mice containing Cre-recombinase knocked into the Rosa26 locus (on a C57Bl/6 background). For this study, heterozygous WMΔ mice were bred to yield wildtype, heterozygous, and homozygous littermates for analyses. Genotyping was by PCR using primer pairs annealing within and outside of the deleted genomic region (for sequences see Table S4). All procedures performed on mice were approved by OHSU IACUC.

### Antibodies

Fibrillin-1 polyclonal antibody (pAb 9543) and monoclonal antibodies (mAb) 15, 78, 201, and 69 have been previously described [9]–[12], [33]. Polyclonal antibody specific for ADAMTSL-6 was generated as described [24]. Antibody to α-smooth muscle actin was purchased from Sigma.

### Cell cultures

CRL2418, a normal dermal fibroblast cell line, was purchased from American Type Culture Collection. WMS fibroblasts were established from a punch biopsy of skin from family member 5010. Explant cultures of P4 mouse skin were established from WMΔ wildtype, heterozygous and homozygous littermates. 1 ml chamber slides were seeded at a density of 200,000 cells/ml and incubated in DMEM, including 10% fetal bovine serum, for 3 to 10 days, as indicated in the figures. Media from the 3-day incubation was collected and stored at −20°C for sandwich ELISAs. Cell layers were analyzed by immunofluorescence.

### Sandwich ELISA

96-well ELISA plates (Corning) were coated with 100 µl of 5 µg/ml streptavidin (Pierce) and incubated overnight at 4°C. Excess streptavidin was removed by extensive washing, and 100 µl/well of 0.25 µg/ml biotinylated monoclonal antibodies (B15 or B201) were incubated at 25°C for 1 hour. After washing, wells were incubated overnight at 4°C with culture medium samples (from 3-day chamber slide cultures of WMS or control fibroblasts). In addition, serially diluted protein standards (rF11) were applied separately to wells coated with biotinylated antibodies and incubated overnight at 4°C. Unbound proteins were removed by washing, and alkaline phosphatase-conjugated monoclonal antibodies (AP201 0.05 µg/ml or AP78 0.5 µg/ml) were incubated in the wells for 1 hour at 25°C. Invitrogen's ELISA amplification system was used for colorimetric detection, according to the manufacturer's protocol. Absorbance was recorded using a Molecular Devices Emax plate spectrophotometer and was then converted to µg/ml, according to standard curve values. Calculations to determine concentration were performed on Excel software.

### Immunofluorescence

Skin was obtained by punch biopsy from a son of family member 5010, following informed consent. Skin was also obtained from WMΔ wildtype and mutant mouse littermates in accordance with OHSU approved IACUC procedures. Immunofluorescence of skin as well as cultured fibroblasts was performed as previously described [10], [11].

### Histology

Histology was performed by the OHSU Histology Core, using standard procedures for Hematoxylin and Eosin and Masson Trichrome stains (Sigma, St. Louis, MO).

### μCT

For μCT analyses, mice were sacrificed at specified time points. μCT measurements and analyses were performed with a Scanco μCT 35 (Scanco Medical, Basserdorf, Switzerland) scanner, according to the manufacturer's instructions.

### Quantitative real-time PCR (qPCR)

qPCR using RNA from WMΔ and wildtype control mouse skin was performed as previously described [11]. Primers for mouse *Col1A1*, *Col1A2*, and *Col3A1* were purchased from SABiosciences (Frederick, MD). The primers for mouse *Periostin* (*Postn*; forward: 5′-catcttcctcagcctccttg-3′; reverse: 5′-tcagaagctccctttcttcg-3′), *Plasminogen activator inhibitor-1* (*Pai1*; forward: 5′-ctttacccctccgagaatcc-3′; reverse: 5′-gacacgccatagggagagaa-3′), and *Connective tissue growth factor* (*Ctgf*; forward: 5′-ctgcctaccgactggaagac-3′; reverse: 5′-ttggtaactcgggtggagat-3′) were individually designed and tested for amplification efficiency.

### Electron microscopy

Immunoelectron microscopy of tissues from WMΔ mouse littermates was performed as described [11]. Tissues were labeled en bloc with anti-fibrillin-1 (pAb 9543) followed by 5 nm secondary gold conjugated antibodies (Amersham Biosciences). Aligned tilt series were acquired from 500 nm thick sections as described [11].

### Expression plasmids

Expression vectors carrying full length human *LTBP-4S* and *LTBP-1S* were kindly provided by Dr. Jorma Keski-Oja and Dr. Daniel Rifkin. The rF90WMΔ and rF84WMΔ expression constructs were cloned from WMS fibroblast cDNA. Full-length *ADAMTSL1* was obtained from human fibroblast cDNA. For cloning of *ADAMTSL2*, a mouse full length cDNA clone (ID RIKEN cDNA F83011122) was obtained. Constructs for *ADAMTSL3* were made using a clone (RIKEN) and mouse lung cDNA. Constructs for *Papilin* were cloned from mouse fibroblast cDNA. A full length human *ADAMTS10* clone (SC309981) was purchased from Origene, and mutations in this clone were corrected.

### Production of recombinant proteins

Generation of recombinant polypeptides representing fragments of LTBP-1 was previously described [13]. The generation of rF90 was described before [16]. All fibrillin and ADAMTSL expression constructs were transfected into 293/EBNA cells for protein expression. All proteins were purified using metal ion affinity chromatography. Protein domain boundaries for the constructs are depicted in Figure 1 and Figure S3.

### Surface plasmon resonance (SPR)

Binding analyses were performed using a BIAcoreX (BIAcore AB, Uppsala, Sweden). Recombinant full length ADAMTSL-1, -2, LTBP-1, -4, and polypeptides ADAMTS-10 C-term, rL1K, rLM, rLN, rF6, rF90, and rF90WMΔ were covalently coupled to CM5 sensor chips (research grade) using the amine coupling kit following the manufacturer's instructions (BIAcore AB). Binding assays were performed at 25°C in 10 mM Hepes buffer, pH 7.4, containing 0.15 M NaCl, 3 mM EDTA, and 0.005% (v/v) P20 surfactant (HBS-EP buffer, BIAcore AB). Kinetic constants were calculated by nonlinear fitting of association and dissociation curves (BIAevaluation 3.0 software). Equilibrium dissociation constants (K_D_) were then calculated as the ratio of kd/ka.

### Pull-down assay

Cell culture media (1 ml) from stably transfected 293/EBNA cells expressing ADAMTS-10 with a C-terminal His_6_-tag, and media from untransfected EBNA cells as a control were adjusted to 20 mM Tris pH 7.8, 5 mM imidazole and incubated for 1 h with 5–20 µg of rF11 (N-terminal half of fibrillin-1, without a His_6_-tag). Subsequently, 50 µl of a 50% Ni-NTA slurry in water was added and incubated for 2 hs. The resin was washed and boiled in 50 µl 1× SDS loading buffer. Eluted proteins were subjected to SDS-PAGE followed by immunoblotting with polyclonal anti fibrillin-1 antibody 9543.

### ELISA assay for active and total TGF-β1

The quantity of TGF-β1 in 100 µl culture medium from confluent fibroblasts (200,000 cells/ml grown for 72 h in 1 ml chamber slides) was determined using the TGF-β1 EMax Immnunoassay kit (Promega). WMS and control fibroblasts were utilized.

### Statistical analysis

Prism 5.02 for Windows (GraphPad, San Diego, CA) was used to perform One-way Analysis of Variance (1-way ANOVA) followed by post-test analysis with Tukey's multiple comparison test. p-values<0.05 were considered significant.

## Supporting Information

Figure S1 Analysis of genomic WMS DNA and mRNA. (a) Southern blot of control (C) and WMS (W) genomic DNA probed with radiolabeled *FBN1* cDNA from exons 8–12. In the WMS DNA, new bands (indicated by arrows) of 6.0 kb (Bsu36I digest) and 3.8 kb (HindIII digest) are observed, along with an apparent reduction of intensity in other bands. (b) RNAse protection assay. Total RNA preparations from control (C) and WMS (W) skin fibroblasts were hybridized to radiolabeled antisense probes from *FBN1* exons 9 and 11 (internal to the deleted region) and exons 21 and 37 (external). The signal intensity of protected internal- and external-region in the control sample showed a ratio of close to 1, indicating that *FBN1* mRNAs were detected equivalently regardless of the probe location. In the WMS RNA, however, the probes internal to the deleted region yielded a signal which was reduced by about 50% relative to probes external to the deletion. Therefore, WMS RNA contains approximately equal amounts of normal and deleted mutant *FBN1* transcripts.
(TIF)

Figure S2 Cross-sections of aortic root from 10-month old wildtype (Fbn1^+/+^), heterozygous (Fbn1^WMΔ/+^) and homozygous (Fbn1^WMΔ/WMΔ^) littermates. Hearts were dissected with the ascending aorta, aortic arch, and a portion of the descending aorta intact to maintain proper orientation. Aortic roots were fixed, cross-sectioned, and stained with toluidine blue. No differences between mutants and wildtype littermates were observed in aortic root morphology, diameter, or wall thickness. Scale bar = 100 µm.
(TIF)

Figure S3 Domain structures and gels showing additional recombinant proteins used in these studies. (a) Domains contained in recombinant papilin and ADAMTSL polypeptides, recombinant ADAMTS-10 polypeptides, and fibrillin-1 polypeptides are depicted schematically. (b) Coomassie stained gels of new recombinant proteins demonstrate the purity of the preparations.
(TIF)

Table S1 Dissociation constants (K_D_) determined using SPR technology. Titrated concentrations of papilin and ADAMTSL molecules (analytes) were injected over immobilized fibrillin-1 peptides (ligands on chip). Full-length ADAMTSL-2 and the C-terminal ADAMTSL-3 polypeptide bind well to wildtype fibrillin-1 peptides but fail to bind to fibrillin-1 peptides containing the WMS deletion. Similarly, binding of papilin fragments suggests interactions with fibrillin-1 that are abolished in a peptide containing the deleted domains.
(DOC)

Table S2 SPR interaction studies between ADAMTSL and LTBP peptides. (a) ADAMTSL-2 interacted with wildtype fibrillin-1 (rF90) but not with mutant rF90 (rF90WMΔ). However, the C-terminal end of LTBP-1 (rL1K) interacted with both wildtype and mutant WMΔ fibrillin-1 peptides. (b) Full-length ADAMTSL-2 failed to interact with the recombinant middle region of LTBP-1 (rL1-M). However, LTBP-1 recombinant C-terminal rL1K interacted with ADAMTSL-2 and -3. Binding was observed between ADAMTSL-3 and rL1M.
(DOC)

Table S3 Specific primers used to detect the deletion in FBN1 cDNA and genomic DNA by PCR.
(DOC)

Table S4 Primers used to determine the genotype of WMΔ mutant mice. Primers anneal within and outside the deleted genomic region.
(DOC)

Video S1 Aligned tilt series of immunolabeled fibrillin-1 microfibrils in wildtype skin. Elastic fiber present in wildtype skin displays periodic labeling of fibrillin microfibrils with pAb 9543. Periodic immunogold labeling emphasizes the organized appearance of wildtype microfibrils.
(WMV)

Video S2 Aligned tilt series of immunolabeled fibrillin-1 microfibrils in mutant WMΔ/WMΔ skin. Elastic fiber present in homozygous mutant WMΔ skin shows much reduced periodicity of fibrillin-1 immunogold labeling, indicating disorganized microfibrils.
(WMV)
