# Supplementary figures and images for: Microenvironmental Regulation by Fibrillin-1
Source: PLoS Genet. 2012 Jan 5;8(1):e1002425. doi: 10.1371/journal.pgen.1002425 (PMC3252277; doi:10.1371/journal.pgen.1002425)

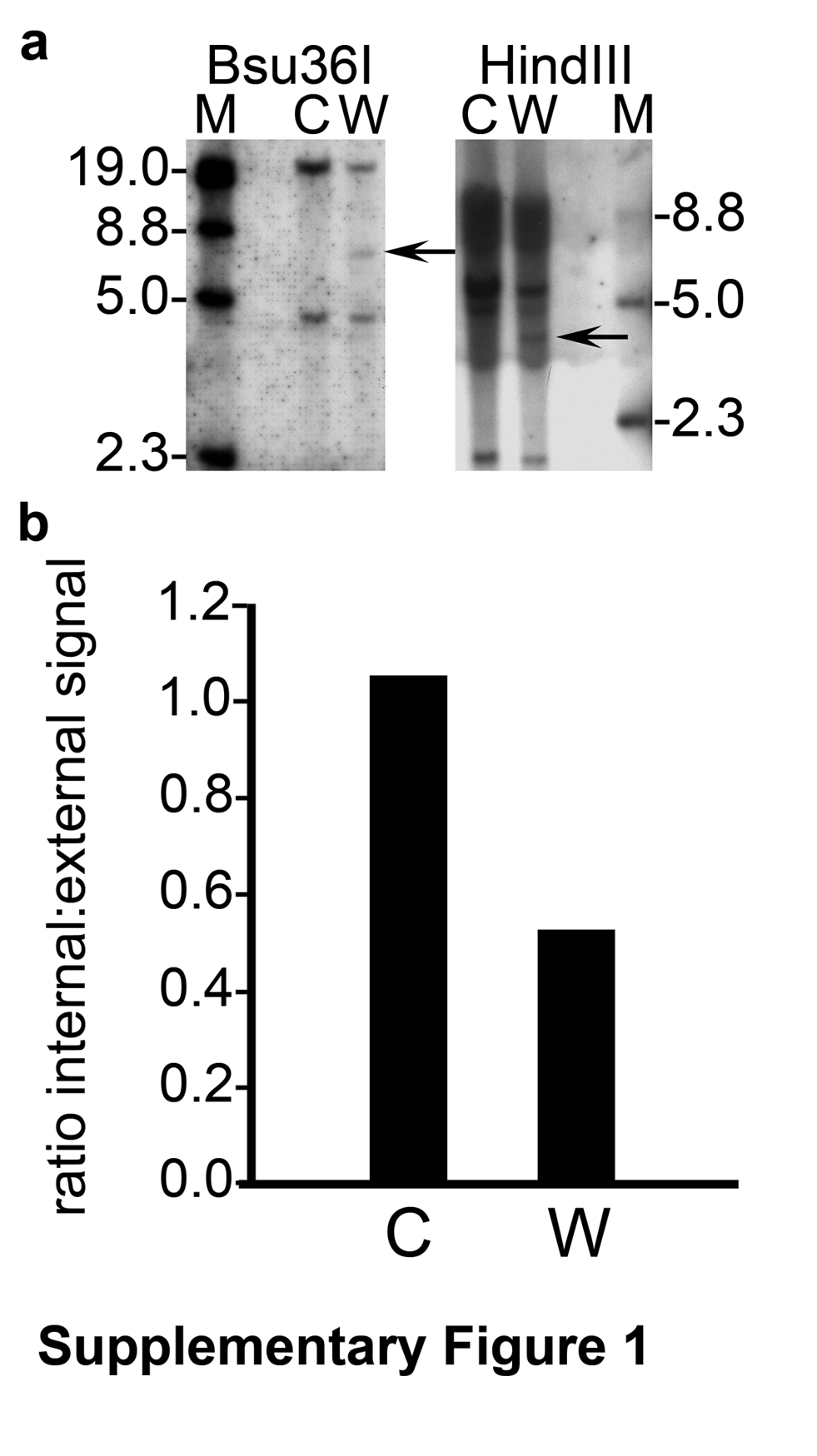

Supplement: Figure S1 — Analysis of genomic WMS DNA and mRNA. (a) Southern blot of control (C) and WMS (W) genomic DNA probed with radiolabeled FBN1 cDNA from exons 8–12. In the WMS DNA, new bands (indicated by arrows) of 6.0 kb (Bsu36I digest) and 3.8 kb (HindIII digest) are observed, along with an apparent reduction of intensity in other bands. (b) RNAse protection assay. Total RNA preparations from control (C) and WMS (W) skin fibroblasts were hybridized to radiolabeled antisense probes from FBN1 exons 9 and 11 (internal to the deleted region) and exons 21 and 37 (external). The signal intensity of protected internal- and external-region in the control sample showed a ratio of close to 1, indicating that FBN1 mRNAs were detected equivalently regardless of the probe location. In the WMS RNA, however, the probes internal to the deleted region yielded a signal which was reduced by about 50% relative to probes external to the deletion. Therefore, WMS RNA contains approximately equal amounts of normal and deleted mutant FBN1 transcripts. (TIF) [file pgen.1002425.s001.tif]

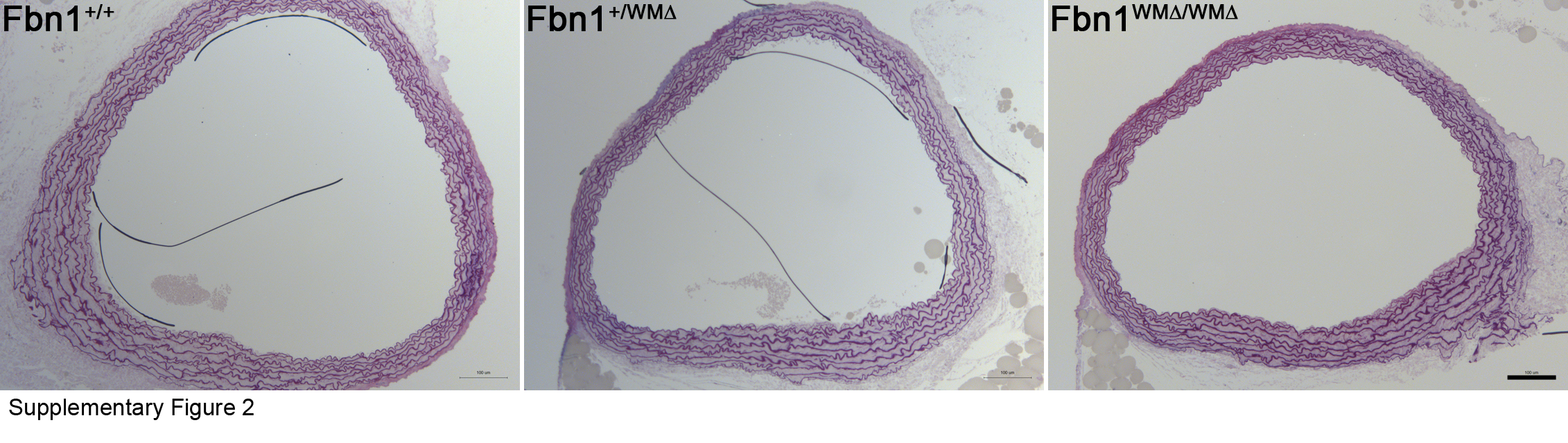

Supplement: Figure S2 — Cross-sections of aortic root from 10-month old wildtype (Fbn1+/+), heterozygous (Fbn1WMΔ/+) and homozygous (Fbn1WMΔ/WMΔ) littermates. Hearts were dissected with the ascending aorta, aortic arch, and a portion of the descending aorta intact to maintain proper orientation. Aortic roots were fixed, cross-sectioned, and stained with toluidine blue. No differences between mutants and wildtype littermates were observed in aortic root morphology, diameter, or wall thickness. Scale bar = 100 µm. (TIF) [file pgen.1002425.s002.tif]

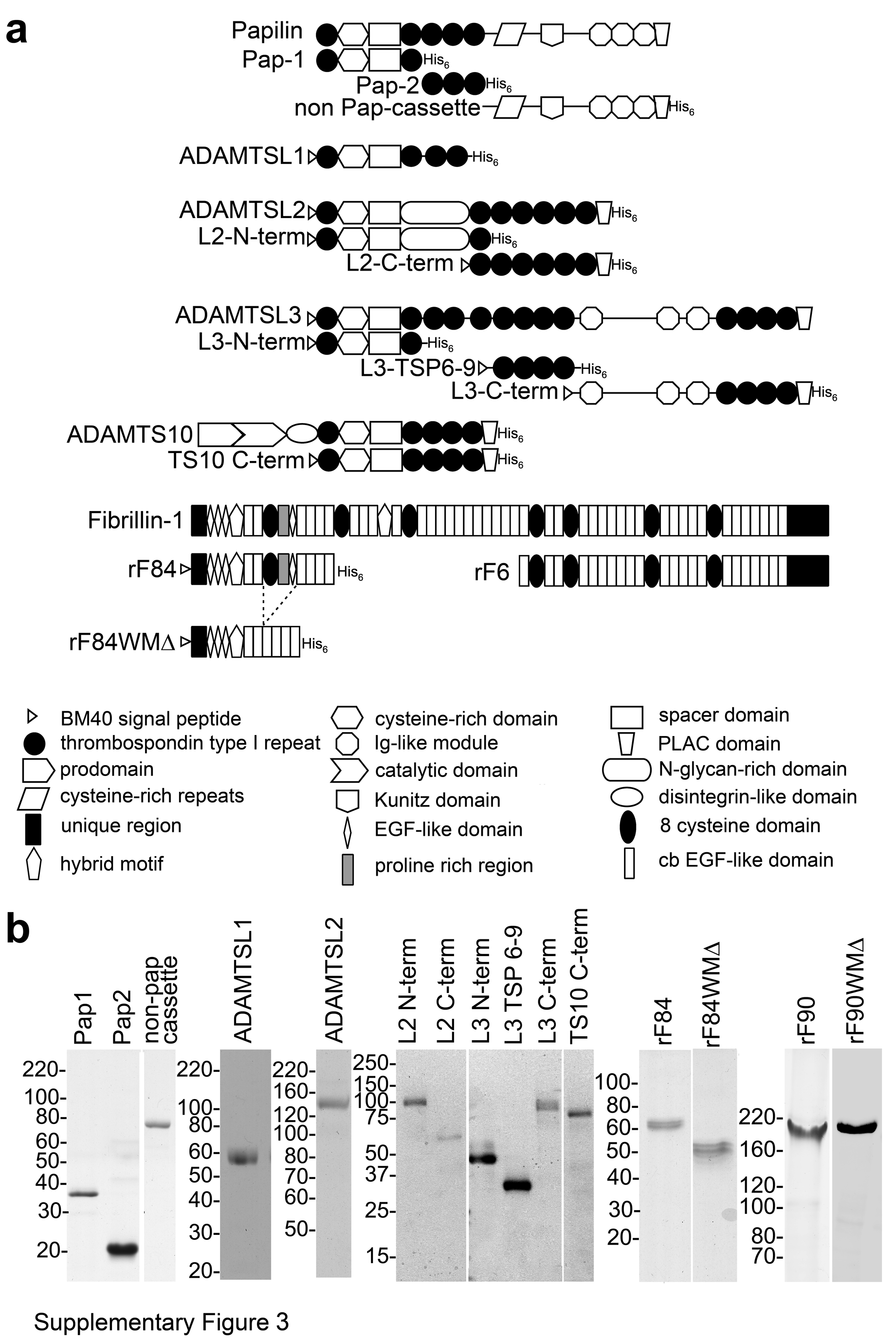

Supplement: Figure S3 — Domain structures and gels showing additional recombinant proteins used in these studies. (a) Domains contained in recombinant papilin and ADAMTSL polypeptides, recombinant ADAMTS-10 polypeptides, and fibrillin-1 polypeptides are depicted schematically. (b) Coomassie stained gels of new recombinant proteins demonstrate the purity of the preparations. (TIF) [file pgen.1002425.s003.tif]
