# Supplementary material for: Microenvironmental Regulation by Fibrillin-1
Source: PLoS Genet. 2012 Jan 5;8(1):e1002425. doi: 10.1371/journal.pgen.1002425 (PMC3252277; doi:10.1371/journal.pgen.1002425)
Supplement: Table S1 — Dissociation constants (KD) determined using SPR technology. Titrated concentrations of papilin and ADAMTSL molecules (analytes) were injected over immobilized fibrillin-1 peptides (ligands on chip). Full-length ADAMTSL-2 and the C-terminal ADAMTSL-3 polypeptide bind well to wildtype fibrillin-1 peptides but fail to bind to fibrillin-1 peptides containing the WMS deletion. Similarly, binding of papilin fragments suggests interactions with fibrillin-1 that are abolished in a peptide containing the deleted domains. (DOC) [file pgen.1002425.s004.doc]

**Table S1**

|  | analyte | analyte | analyte | analyte | analyte | analyte | analyte | analyte |
| --- | --- | --- | --- | --- | --- | --- | --- | --- |
| Ligand on chip | ADAM  TSL1 | ADAM  TSL2 | ADAM  TSL2 N-term | ADAM  TSL2 C-term | ADAM  TSL3 C-term | Pap1 | Pap2 | non Pap  cassette |
| rF90 | nb | 200nM | 2.5μM | 1.2 μM | 6nM | nd | nd | nd |
| rF90WMΔ | nb | nb | nb | nb | nb | nd | nd | nd |
| rF84 | nd | 96nM | nd | nd | nd | 1.5μM | 850nM | 380nM |
| rF84WMΔ | nd | nb | nd | nd | nd | nb | nb | nb |
| rF6 | nb | nb | nb | nd | nb | nd | nd | nd |

nb: no binding; nd: not determined.
