# Supplementary material for: Microenvironmental Regulation by Fibrillin-1
Source: PLoS Genet. 2012 Jan 5;8(1):e1002425. doi: 10.1371/journal.pgen.1002425 (PMC3252277; doi:10.1371/journal.pgen.1002425)
Supplement: Table S2 — SPR interaction studies between ADAMTSL and LTBP peptides. (a) ADAMTSL-2 interacted with wildtype fibrillin-1 (rF90) but not with mutant rF90 (rF90WMΔ). However, the C-terminal end of LTBP-1 (rL1K) interacted with both wildtype and mutant WMΔ fibrillin-1 peptides. (b) Full-length ADAMTSL-2 failed to interact with the recombinant middle region of LTBP-1 (rL1-M). However, LTBP-1 recombinant C-terminal rL1K interacted with ADAMTSL-2 and -3. Binding was observed between ADAMTSL-3 and rL1M. (DOC) [file pgen.1002425.s005.doc]

Table S2

(**a**)

|  | analyte | analyte | analyte | analyte |
| --- | --- | --- | --- | --- |
| Ligand on chip | rL1M | rL1K | rF90 | rF90WMΔ |
| ADAMTSL-2 | nb | nb | + | - |
| rL1K | nd | nd | + | + |

(**b**)

|  | analyte | analyte | analyte | analyte |
| --- | --- | --- | --- | --- |
| Ligand on chip | ADAM  TSL1 | ADAM  TSL2 | ADAM  TSL2 N-term | ADAM  TSL3 C-term |
| LTBP4 | nb | nb | nb | nb |
| rL1N | nd | nd | nb | nd |
| rL1M | nb | nb | nb | + |
| rL1K | nd | nd | + | + |

+: binding; nb: no binding; nd: not determined.
