# Supplementary material for: Microenvironmental Regulation by Fibrillin-1
Source: PLoS Genet. 2012 Jan 5;8(1):e1002425. doi: 10.1371/journal.pgen.1002425 (PMC3252277; doi:10.1371/journal.pgen.1002425)
Supplement: Table S3 — Specific primers used to detect the deletion in FBN1 cDNA and genomic DNA by PCR. (DOC) [file pgen.1002425.s006.doc]

Table S3

| designation | sequence | position |
| --- | --- | --- |
| N1929S | 5’-CTCTGTCAGGGAGGAAATTGC-3’ | exon 7, FBN1 766-786 |
| + N981AS | 5’-CTTCACAGTTCTTCCCATCTCG-3’ | exon 13, FBN1 1714-1693 |
| FBN1-Int8.2S | 5’-ATCAGTGGTCCCCAACCTT-3’ | IVS8 -1667 |
| + FBN1E12AS | 5’-CAGTTAGCATATATGTCCCAC-3’ | IVS12+48 |
