# Supplementary material for: Microenvironmental Regulation by Fibrillin-1
Source: PLoS Genet. 2012 Jan 5;8(1):e1002425. doi: 10.1371/journal.pgen.1002425 (PMC3252277; doi:10.1371/journal.pgen.1002425)
Supplement: Table S4 — Primers used to determine the genotype of WMΔ mutant mice. Primers anneal within and outside the deleted genomic region. (DOC) [file pgen.1002425.s007.doc]

Table S4

| designation | sequence | products |
| --- | --- | --- |
| P892_77 | 5’-GGCTGTCTTTAATCAGAGATGCGTC-3’ | 6181 bp for wt, |
| P892_78 | 5’-GGGTTTCAAATGCTCAAGTCACGC-3’ | 402 bp for het and hom |
| P892_79 | 5’-AGGGTGAGGCATTGGAGGTATTGAG-3’ | 520 bp for wt and het |
| P892_80 | 5’-CAAAAATGGGACTTGGCAGAAGGAAG-3’ | no band for hom |
